# Synthesis of armchair graphene nanoribbons from the 10,10′-dibromo-9,9′-bianthracene molecules on Ag(111): the role of organometallic intermediates

**DOI:** 10.1038/s41598-018-21704-3

**Published:** 2018-02-22

**Authors:** K. A. Simonov, A. V. Generalov, A. S. Vinogradov, G. I. Svirskiy, A. A. Cafolla, C. McGuinness, T. Taketsugu, A. Lyalin, N. Mårtensson, A. B. Preobrajenski

**Affiliations:** 1 Department of Physics and Astronomy, Uppsala University, Box 516, 75120 Uppsala, Sweden; 2 MAX IV Laboratory, Lund University, Box 118, 22100 Lund, Sweden; 3 V.A. Fock Institute of Physics, St. Petersburg State University, 198504 St. Petersburg, Russia; 4 School of Physical Sciences, Dublin City University, Dublin, D09 Ireland; 5 School of Physics, Trinity College Dublin, College Green, Dublin, D02 Ireland; 6 Department of Chemistry, Faculty of Science, Hokkaido University, Sapporo, 060-0810 Japan; 7 Global Research Center for Environment and Energy Based on Nanomaterials Science (GREEN), National Institute for Materials Science (NIMS), Tsukuba, 305-0044 Japan

## Abstract

We investigate the bottom-up growth of N = 7 armchair graphene nanoribbons (7-AGNRs) from the 10,10′-dibromo-9,9′-bianthracene (DBBA) molecules on Ag(111) with the focus on the role of the organometallic (OM) intermediates. It is demonstrated that DBBA molecules on Ag(111) are partially debrominated at room temperature and lose all bromine atoms at elevated temperatures. Similar to DBBA on Cu(111), debrominated molecules form OM chains on Ag(111). Nevertheless, in contrast with the Cu(111) substrate, formation of polyanthracene chains from OM intermediates *via* an Ullmann-type reaction is feasible on Ag(111). Cleavage of C–Ag bonds occurs before the thermal threshold for the surface-catalyzed activation of C–H bonds on Ag(111) is reached, while on Cu(111) activation of C–H bonds occurs in parallel with the cleavage of the stronger C–Cu bonds. Consequently, while OM intermediates obstruct the Ullmann reaction between DBBA molecules on the Cu(111) substrate, they are required for the formation of polyanthracene chains on Ag(111). If the Ullmann-type reaction on Ag(111) is inhibited, heating of the OM chains produces nanographenes instead. Heating of the polyanthracene chains produces 7-AGNRs, while heating of nanographenes causes the formation of the disordered structures with the possible admixture of short GNRs.

## Introduction

Recently the on-surface bottom-up synthesis of covalent nanostructures from constituent building blocks had developed into a very active research field^1–7^. Among the various classes of covalently-bonded nanostructures graphene nanoribbons (GNRs) are particularly interesting. Due to the quantum confinement and edge effects these ultra-narrow graphene stripes appear as promising candidates for low-dimensional electronic devices, but only if atomic precision in their edge geometry is achieved^8–14^. This strong dependence of electronic properties on the atomic structure has generated increased attention to the bottom-up fabrication of GNRs^7,15–38^.

One of the most successful strategies for growth of GNRs utilises metal-catalysed coupling of halogen-substituted molecules, also known as Ullmann-type coupling reaction^39–54^. Being part of a multistep process, the Ullmann-type reaction between specifically designed molecules is aimed to result in the formation of polymerized molecular chains, which can be transformed to atomically precise graphene nanoribbons upon further cyclodehydrogenation at higher temperatures^7,15^. In the majority of the studies this method was used for the preparation of GNRs with various atomic structures on Au(111), Au(110) or Au(788) substrates^7,15–29,38^. In particular, the most widely studied nanoribbon is that with armchair edges and a width (N) of seven carbon atoms (7-AGNR) that grows from the 10,10′-dibromo-9,9′-bianthracene (DBBA)^15–20,23,25,29^.

The bottom-up growth of GNRs on the less inert noble metals Ag(111)^15,18,35^ and Cu(111)^30–34,36,37^ was also demonstrated. Surprisingly, it turned out that, unlike for many other precursor molecules^41–44,50–52^, polymerization of DBBA on Cu(111) does not proceed *via* the Ullmann-type reaction^32,33,36,37^. Initially we argued against this scenario^33^, but it has recently been confirmed by non-contact AFM experiments^35,36^. It was shown that instead of the Ullmann coupling, a site-selective surface-assisted dehydrogenative covalent coupling reaction between molecular units takes place on Cu(111). As a result, annealing of DBBA on a Cu(111) substrate yields chiral (3,1)-GNRs and no 7-AGNRs are formed^32,33,36,37^. Similar to Cu(111), surface-assisted dehydrogenation reactions followed by the formation of covalent C–C intermolecular connections were demonstrated to be feasible on Ag(111)^55,56^. At the same time on-surface reaction with DBBA on Ag(111) yields 7-AGNRs^15,18^. Despite having demonstrated the formation of 7-AGNRs on Ag(111) at the outset of the revolution in bottom-up fabrication of GNRs, the growth mechanism of 7-AGNRs from DBBA molecules on Ag(111) has not been as actively studied as that on Au(111) and Cu(111). Instead this mechanism has always been implicitly accepted to be the same as that for 7-AGNR formation on the Au(111) surface^35^. Nevertheless, the results of the two most recent studies of DBBA molecules on the Ag(111) surface imply that the reaction pathway for GNR formation on Ag(111) is different from that on Au(111)^18,57^. In particular, Ullmann-type formation of covalent chains has not been observed for DBBA on Ag(111). On the other hand annealing of DBBA on Ag(111) at 180 °C is reported to yield flat nanographene units, similar to those formed on Cu(110)^58^ and Au(111)^59^ surfaces from DBBA and its chloro-analogue, respectively. However, the nanographenes on Cu(110) and Au(111) do not form GNRs upon annealing, giving rise to highly-branched structures instead^58,59^. Therefore the exact reaction mechanism for the formation of polyanthracene precursors for 7-AGNRs on Ag(111) still remains to be understood.

It is known that on copper and silver surfaces the dehalogenated precursors usually tend to assemble into partly covalent organometallic (OM) intermediates prior to purely covalent C–C coupling. These intermediate structures are composed of molecular units linked through the C–M–C bonds^41–47,50–53,60^. In particular, OM chains were observed on Cu(111) surface after deposition of DBBA molecules at room temperature (RT) followed by a complete debromination of DBBA^34,58,61^. Nevertheless, to date the role of OM intermediates in the formation of 7-AGNRs on Ag(111) from DBBA precursors has not been studied explicitly. According to previous studies^45,50^, the C–Ag–C bonds have a higher probability than the C–Cu–C bonds of being directly converted into covalent C–C bonds (without exceeding the thermal stability of the molecular units). Hence additional information about the OM intermediates and conditions for the conversion of the OM bond to covalent-bonds can shed light on the reasons for different reaction pathways for DBBA on Ag(111) and Cu(111).

In this investigation we have used finely-tuned protocols to investigate the mechanism of GNR formation from DBBA precursor molecules on an Ag(111) substrate. Key reaction steps were compared with the analogous process on the Cu(111) surface. In particular we were interested in identifying i) the possible existence of any DBBA-based OM intermediates on Ag(111); ii) the structure of these intermediates; iii) the temperature regimes and mechanism for the formation of covalent chains or nanographenes on Ag(111) and iv) the possible role of the OM intermediates in the observed differences in the reaction pathways for DBBA on Ag(111) and Cu(111). To address these questions we have performed a study of DBBA adsorption and transformation on the Ag(111) surface by using X-ray photoelectron spectroscopy (XPS) and Scanning Tunnelling Microscopy (STM) in combination with density functional theory (DFT) calculations.

We demonstrate that growth of GNRs on the Ag(111) from DBBA precursors includes the formation of OM chains. Unlike the analogous chains formed by DBBA on Cu(111) surface, OM chains on Ag(111) can be directly converted into the covalent polyanthracene chains, which play the role of a polymer precursor for the 7-AGNRs. As an alternative to the Ullmann-type reaction, nanographene units can be grown instead of polyanthracene chains, but 7-AGNRs cannot be formed in this case. It is shown that the strength of the C–M bonds plays an important role in the reaction pathway for DBBA on Ag(111) and Cu(111).

## Results

**Figure 1:**
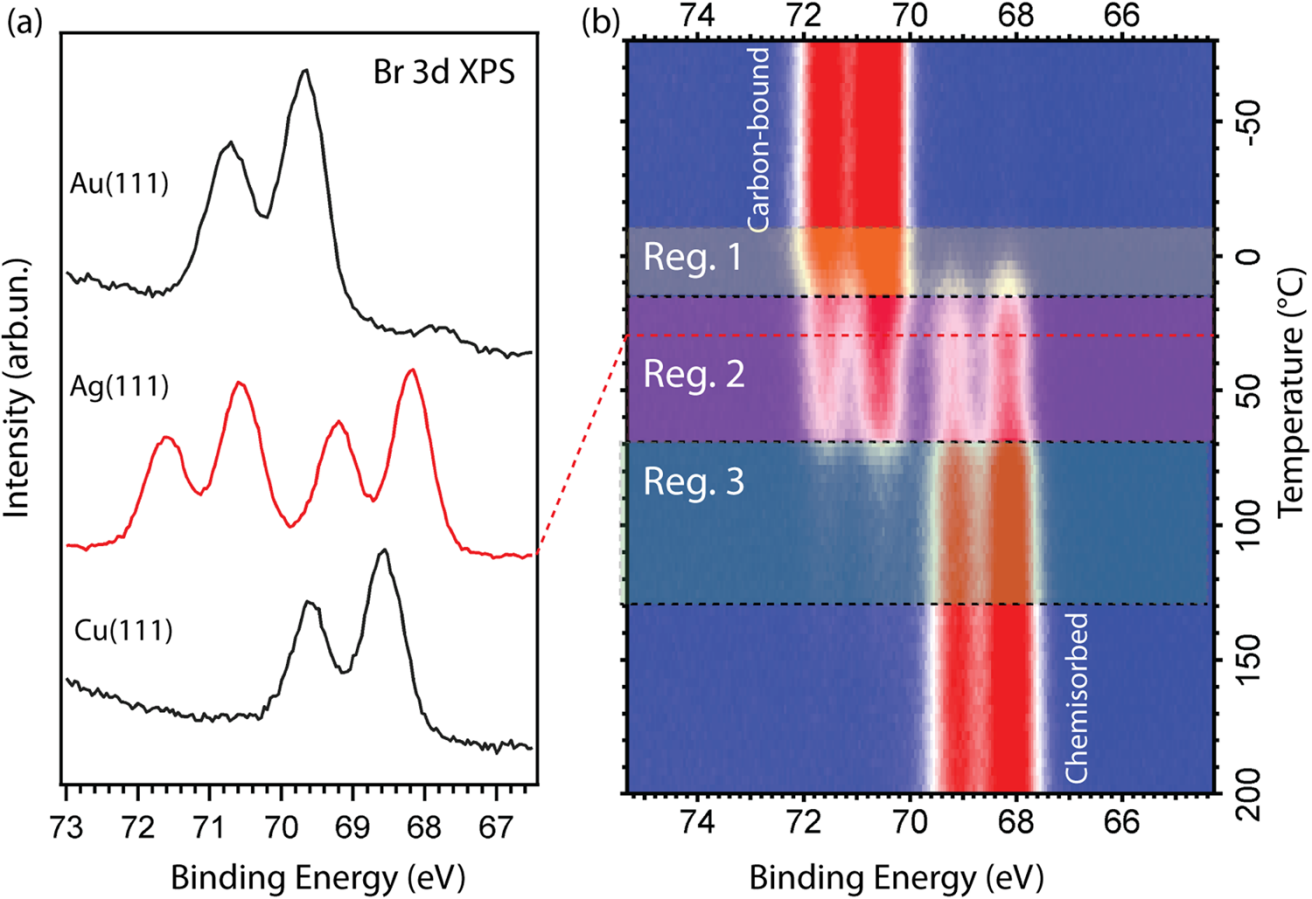
(**a**) Comparison of the RT Br 3d XPS spectra from DBBA molecule on substrates with different reactivity: Au(111), Ag(111) and Cu(111). (**b**) Evolution of the Br 3d XPS spectrum for DBBA/Ag(111) mapped as a function of increasing sample temperature. The temperature varies from −80 °C to 200 °C. The red dashed line marks the spectrum corresponding to RT. Three regions, Reg.1, Reg.2 and Reg.3, indicate different stages of the DBBA debromination process on Ag(111) (see text for more information). The photon energy is 170 eV. For (**b**) the same color scale was used at all temperatures, spectra were normalised to the ring current.

The cleavage mechanism of the Br side groups can play a decisive role in the covalent coupling of the precursor molecules^30,31,45,49,50,58,59^. Therefore a detailed investigation of the debromination of DBBA on Ag(111) is a good starting point for understanding the on-surface reaction mechanism. The dissociation of the C–Br bond at RT is well documented for various brominated precursors, and is expected to be complete on Cu(111)^30,31,49,50,58^, partial on Ag(111)^45,49,50^ and absent on Au(111)^1,30,31,52^. As a consequence the Br 3d XPS spectra for DBBA on Au(111), Ag(111) and Cu(111) measured at RT differ considerably (Fig. 1a). In agreement with our previous studies of DBBA adsorption on metal surfaces at RT^30,31,58^, the Br 3d XPS spectra of both DBBA on Au(111) and DBBA on Cu(111) consist of one spin-orbit doublet, although at considerably different binding energies (E_B_). For DBBA on Au(111) the binding energy E_B_ (Br 3d_5/2_) = 69.7 eV corresponds to a carbon-bound bromine atom while for DBBA on Cu(111) the spin-orbit component at E_B_ (Br 3d_5/2_) = 68.5 eV is produced by atomic Br adsorbed on the Cu(111) surface^30,31^. On the other hand, the low- and high-binding energy spin-doublets for DBBA on Ag(111) are present simultaneously in the spectrum at RT in approximately equal intensities. The presence of two components confirms the coexistence of carbon-bound (E_B_ (Br 3d_5/2_) = 70.6 eV) and chemisorbed (E_B_ (Br 3d_5/2_) = 68.2 eV) bromine for DBBA on Ag(111)^45^.

In order to obtain a detailed insight into the process of DBBA debromination on Ag(111) the molecules were deposited on Ag(111) at −150 °C and the Br 3d XPS spectrum was recorded as a function of increasing sample temperature (with a heating rate of 3 °C/min). As shown in Fig. 1(b), up to −10 °C the Br 3d XPS spectrum of DBBA on Ag(111) consists of only one spin-doublet component which originates from the carbon-bound Br thus indicating that DBBA molecules stay intact on the Ag(111) surface at low temperatures. In contrast, at temperatures exceeding 130 °C only the signal from chemisorbed bromine is observed in the Br 3d XPS spectrum. Therefore the debromination process of DBBA on Ag(111) occurs in a large temperature interval from −10 °C to 130 °C, which can be further divided into three regions (Reg.1, Reg.2 and Reg.3) as indicated in Fig. 1(b). In Reg.1 (from −10 °C to +15 °C) a partial debromination of DBBA/Ag(111) takes place. As a consequence, a second Br 3d component, corresponding to chemisorbed bromine appears, causing a redistribution of the total Br 3d intensity from the high-E_B_ to the low-E_B_ spin-doublet.

Starting at 15 °C and up to 70 °C (Reg.2) the Br 3d XPS spectrum consists of two spin-doublet components with almost equal intensity between 25 °C and 60 °C. Above 70 °C the high-E_B_ component reduces to zero intensity (Reg.3). It is important to note that the total summed intensity of all of the Br 3d line components remains almost constant in the entire studied temperature interval, instead being redistributed between the different components related to carbon-bound bromine or chemisorbed bromine on the Ag surface. Desorption of the chemisorbed Br starts around 250 °C and is completed at 350 °C (not shown).

The described character of the DBBA debromination dynamics on Ag(111) differs significantly from that previously studied on Au(111)^30,31^ and Cu(111)^30,31,58^, where both Br atoms dissociate from the DBBA molecules simultaneously. On the contrary, it resembles debromination of DBBA on Cu(110)^58^, where the DBBA molecule retains a single Br atom over a wide temperature range. From the Br 3d XPS temperature map data we identify the following most interesting temperature intervals for closer consideration: (i) 20–40 °C, where molecules are partially debrominated; (ii) 100–130 °C, where the debromination is complete and the formation of OM intermediates is anticipated; and (iii) 130–200 °C, where, according to the previous studies of on-surface reactions on Ag(111)^45,47,50^, the conversion of OM bonds into the covalent bonds is possible.

Two co-existing self-assembled structures formed by DBBA molecules on Ag(111) at RT are illustrated in Fig. S1(a–c) in the Electronic Supplementary Information (ESI). Recently, the same structures have been reported by Shen *et al*.^57^ and interpreted as a result of the self-assembly of intact DBBA molecules mainly governed by the intermolecular interactions. Notably, no netlike islands of “armchairlike molecular chains” reported in ref.^57^ were observed by us at RT. At the same time only such conglomerates of “molecular chains” were observed after deposition of DBBA on the Ag(111) substrate kept at 100 °C, as shown in Fig. S2(a–d) for several different coverages. The islands of “molecular chains” can be seen not only at the coverages below 0.4 monolayer (as it is suggested in ref.^57^), but also at surface coverages close to, or even higher than a monolayer. Moreover, “molecular chains” never co-exist with other ordered molecular structures. Additionally our STM measurements (Fig. S3) show that self-assembled structures characteristic for DBBA on Ag(111) deposited at RT can be completely transformed into the “molecular chains” by heating. As will be discussed later, these “molecular chains” are composed of completely debrominated DBBA molecules. Therefore their presence at RT in ref.^57^ can be explained by the complete debromination of some fraction of DBBA in experiments as was reported for 2,2′-dibromo-9,9′-bianthracene molecule on Ag(111)^35^.

Unlike 2,2′-dibromo-9,9′-bianthracene molecules on Ag(111)^35^, for DBBA on Ag(111) at RT ordered structures do not co-exist with the large arrangements of disordered adsorbates with significantly larger apparent height, which were interpreted in ref.^35^ as arrays of intact 2,2′-dibromo-9,9′-bianthracene molecules This implies that the presence of Br 3d XPS signals from both chemisorbed Br atoms on the Ag (111) surface and Br atoms bonded to the DBBA in Reg.2 is due to partial debromination of the precursor molecules, and not due to the co-existence of both islands with fully debrominated and intact DBBA molecules. Therefore, while the previous study considered all DBBA molecules at RT to be intact^57^, partial debromination must be taken into account when analysing the adsorption of DBBA on Ag(111) at RT. Following the conclusion drawn previously for DBBA on Cu(110)^58^, we suggest that the partial debromination of DBBA on Ag(111) results in a tilted adsorption geometry of the molecular fragments, with one of the anthracene subunits bonded to the surface and the second pointing upwards thus increasing the distance between the remaining Br atom and the surface (in a “one-legged” configuration). This is in agreement with Fig. S1(a–c) and with the conclusions of ref.^57^, which show that the arrangement of individual molecules and molecular domains is influenced by the interaction with the silver substrate. A more detailed analysis of the STM images of DBBA on Ag(111) at RT that are presented in Figs S1 and S3 remains beyond the scope of this work.

**Figure 2:**
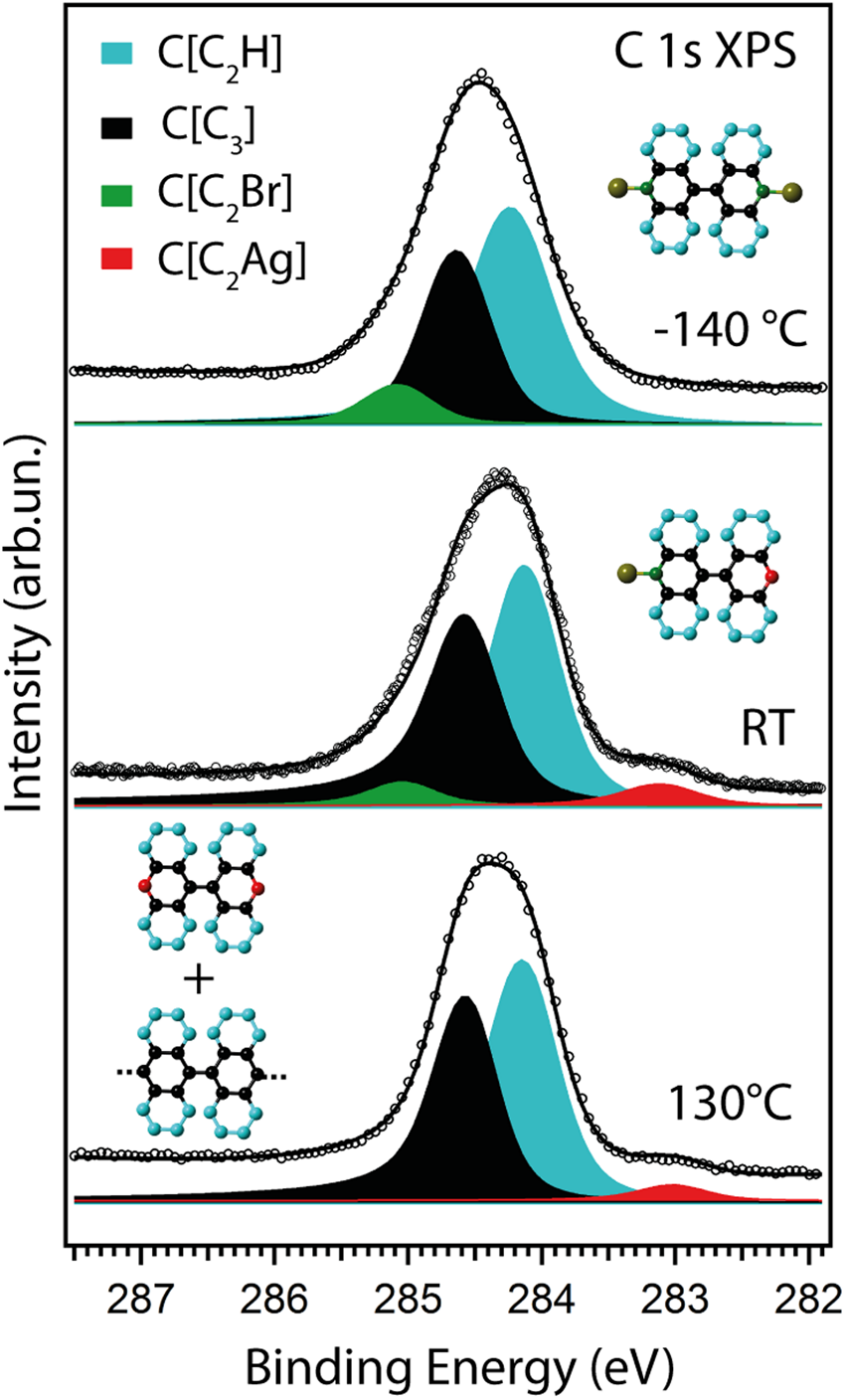
C 1s XPS spectra for DBBA/Ag(111) at −140 °C (top), at RT (middle) and after annealing up to 130 °C (bottom). Corresponding structural models are shown as insets. The photon energy is 380 eV.

The C 1s XPS spectra measured directly after deposition at −140 °C, at RT and after annealing up to 130 °C (Fig. 2) were acquired in order to track changes in the chemical state of the carbon atoms within the DBBA molecule. The photoemission spectra are centred at a binding energy of E_B_ = 284.3–284. 5 eV and have a similar overall shape. Analysis of the evolution of the C 1s line shape for DBBA on Ag(111) was performed using the fitting procedure, successfully implemented previously for the investigation of DBBA on Au(111), DBBA on Cu(111) and DBBA on Cu(110)^29,30,31,58^. By analogy with the C 1s XPS spectrum of intact DBBA on Au(111) at RT, the C 1s XPS spectrum of DBBA on Ag(111) at low temperatures can be fitted with three components, (indicated as black, cyan and green components in Fig. 2), associated with the three inequivalent groups of carbon atoms, correspondingly colored, in the structural model shown in the inset in Fig. 2. The relative intensities of the components thus should match the corresponding fraction of black-colored C atoms with three neighbouring carbons (C[C_3_] sites); cyan-colored hydrogen-bonded C atoms at the edges of the molecule (C[C_2_H] sites); and green-colored C atoms bound to Br (C[C_2_Br] sites). The relative peak intensities derived from the peak-fit analysis and the fractions of carbon atoms in the corresponding sites are compared in Table 1.

**Table 1:** Results of the peak-fit analysis performed in Fig. 2: comparison of the ratio between the number of carbon atoms in C[C_3_], C[C_2_Br] and C[C_2_Ag] sites and the number of carbon atoms in the C [C_2_H] sites.

Sites	−140 °C		RT		130 °C	
	model	fit	model	fit	model	fit
C[C_3_]	0.63	0.64	0.63	0.63	0.63	0.76
C[C_2_H]	1	1	1	1	1	1
C[C_2_Br]	0.13	0.14	0.06	0.07	0	0
C[C_2_Ag]	0	0	0.06	0.07	0.13	0.06

The number of carbon atoms in the C [C_2_H] sites is taken as a reference since, according to the structural models proposed in Fig. 2, it should remain unchanged upon annealing. At 130 °C the fit deviates from the model of debrominated DBBA molecule linked to two Ag atoms. This can be explained by the onset of the formation of covalent C–C bonds. See text for more explanation.

To account for the fact that the DBBA molecules are half-debrominated on Ag(111) at RT, an additional component located at a binding energy of 283.1 eV is included in the fitting procedure withdrawing half of the intensity from the high-E_B_ C[C_2_Br] component at 285.1 eV. This new low-E_B_ component is attributed to the C–Ag bond formed between the debrominated carbon atom (red-colored atom in the structural model) and the silver surface (C[C_2_Ag] sites). A similar low-E_B_ component of the C 1s XPS line was previously observed for debrominated DBBA on Cu(111)^30,31^, Cu(110)^58^, and for tetrathienoanthracene on Ag(111)^50^ indicating carbon-metal bonding. Therefore our spectroscopic data confirm that at RT one of two anthracene subunits in the DBBA molecule is directly anchored to the silver surface.

Annealing to 130 °C, which was chosen as it is the highest temperature of interval (ii), eliminates the asymmetry on the high-E_B_ side of the C 1s line. This change in the line shape can be correlated with the disappearance of the high-E_B_ C[C_2_Br] component in the Br 3d XPS data due to the complete debromination of the DBBA molecules as evident from Fig. 1(b). The low-E_B_ component can then be attributed to carbon atoms in the C[C_2_Ag] sites as in the RT data. In order to match the corresponding structural model (shown to the top–left in the bottom spectrum, Fig. 2), the intensity of the C[C_2_Ag] component (shown in red), should be equal to twice that of the corresponding component at RT. However, as can be seen from Table 1, the intensity of this component remains almost unchanged. This can be explained by the onset of the formation of covalent C–C bonds at 130 °C, instead of organometallic bonds, which account for the slightly increased relative intensity of the C[C_3_] component.

Guided by the XPS data, it is proposed that the formation of OM chains on Ag(111) can be expected to occur at approximately 120 °C. This temperature is high enough to activate complete debromination of the DBBA units, but is insufficient for a conversion of the OM intermediates into covalent products^45,47,50^.

**Figure 3:**
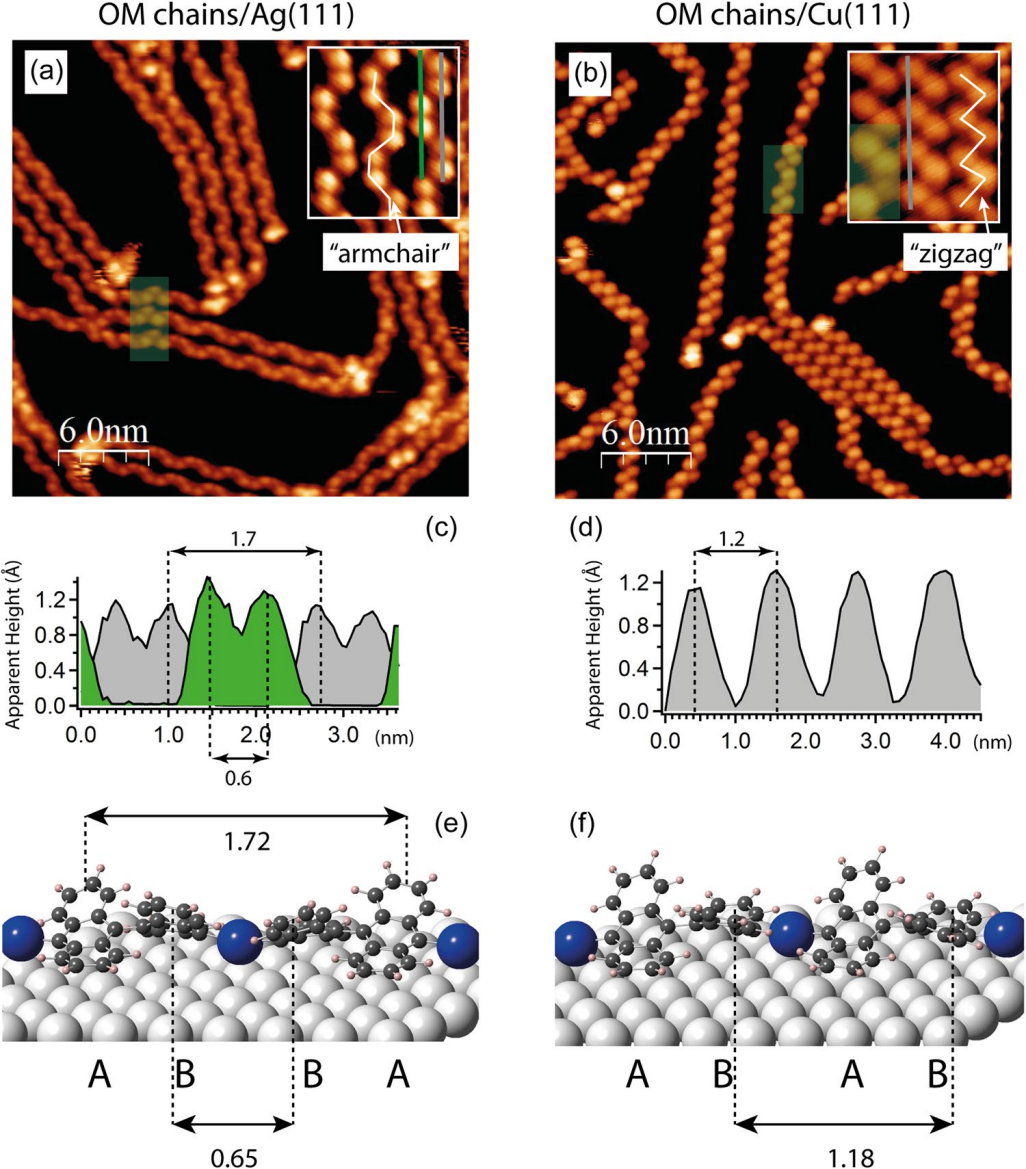
STM images showing OM chains obtained after 30 min annealing of DBBA/Ag(111) at 120 °C (**a**); and OM chains which are formed by DBBA on Cu(111) at RT (**b**). The insets show zoomed-in areas with three OM chains. The “armchair” and “zigzag” chain patterns are highlighted by the white line. The semi-transparent green rectangles highlight sample areas with structural defects in the OM chains. (**c**) and (**d**) are the line profiles taken along the lines of the corresponding color in the insets for OM chain on Ag(111) and Cu(111), accordingly. DFT-optimized models for OM chain on Ag(111) (**e**) and Cu(111) (**f**) are shown, where characteristic distances are marked for comparison with the experimental line profiles. ABBA and ABAB stand for the periodicity characteristic for the “armchair” and “zigzag” OM chains. In the models, the light-grey colored atoms are the substrate atoms, blue colored atoms are the metal adatoms involved in the formation of the OM chain, dark-grey colored atoms are carbon atoms, and light-pink colored atoms are hydrogen atoms. All distances are shown in nm. Tunneling parameters (V_S_/I_T_): (**a**) -1.3 V/300 pA; (**b**) -1.5 V/150 pA.

Figure 3(a) and (b) show STM images of DBBA on Ag(111) after annealing to 120 °C and, for comparison, “molecular chains” formed by DBBA on Cu(111) at RT, respectively. According to our previous STM experiments^34,58^, recently supported by extensive studies by means of nc-AFM and high-speed STM^61^, DBBA molecules form OM chains on Cu(111) at RT. These chains are composed of fully debrominated DBBA molecules linked via the Cu atom. Individual chains tend to assemble into OM islands^61^. Similar regions of “chain-like” structures are clearly visible on Ag(111). By analogy with previous studies of halogen-substituted molecules on Ag(111)^45–47,50^, it is proposed that at 120 °C debrominated DBBA molecules can be coupled into OM intermediates *via* the Ag adatoms. Enlarged images of three individual OM chains on Ag(111) and Cu(111) are shown in the insets in Fig. 3(a) and (b). In agreement with other STM studies^15,34–37,58,61^,the bright protrusions, which appear in the STM image on both sides of the chain axis, are associated with the upward-pointing lobes of the anthracene subunits, constituting the DBBA molecule. On Ag(111) these bright protrusions form an “armchair” pattern (highlighted by the white line shown in the inset in Fig. 3a), which is different from a “zigzag” pattern characteristic for the OM chains on Cu(111) seen in Fig. 3(b).

Line profiles measured along the lines shown in the inset images are illustrated in Fig. 3(c) and (d) together with the characteristic distances. According to the model for the OM chain on Cu(111)^34,58,61^, each of the DBBA radicals in the chain is bridged between two metal adatoms and the anthracene lobes are tilted in an alternating fashion with respect to each other. Similar structures are suggested for the OM chains on Ag(111), but in order to produce the “armchair” pattern observed in the STM images, the two anthracene lobes located next to each other on the same side of the chain’s axis should be tilted in the same direction. By analogy with OM chains on Cu(111)^61^, the bridging Ag atoms for OM chains on Ag(111) are expected to sit closer to the substrate than the protruding molecular lobes and therefore are not visible in the STM images^61,62^.

The DFT-optimized geometries of the OM chains on Ag(111) and Cu(111), are shown in Fig. 3(e) and (f), respectively. These structures were obtained by optimization of OM chains having different orientation, position, and periodicity with respect to each other on the metal surface as described in the Computational Details. The resulting structures are in agreement with the STM images–the OM chain on Ag(111) is characterized by the ABBA periodicity (see figure caption for details), while the OM chain on Cu(111) is of ABAB type. Notably, in case of irregularities in the chain due to the tilt, kink, or missing molecule, short sections with ABAB or ABBA periodicity can get incorporated into the OM chains on both Ag(111) and Cu(111), respectively. Some areas with these structures are highlighted by the semi-transparent rectangles in Fig. 3(a,b). This type of defects is more frequently observed on Cu(111) where, according to the DFT calculations, the energy difference between ABBA and ABAB structures is only 0.04 eV. In comparison, the energy difference between the less stable “zigzag” (ABAB) and “armchair” (ABBA) chains on Ag(111) is 0.15 eV. It should be noted that it is not possible to exclude the participation of substrate metal atoms in the formation of OM chains instead of the native adatoms. Nevertheless, as was noted above, this type of bonding was observed previously^45–47,50,61^ and better reproduces our experimental results. Interestingly, by analogy with OM chains on Cu(111)^34,58,61^, the OM chains on Ag(111) are further stabilized via the formation of 2D islands, which are seen clearly in Fig. S2(a,b).

**Figure 4:**
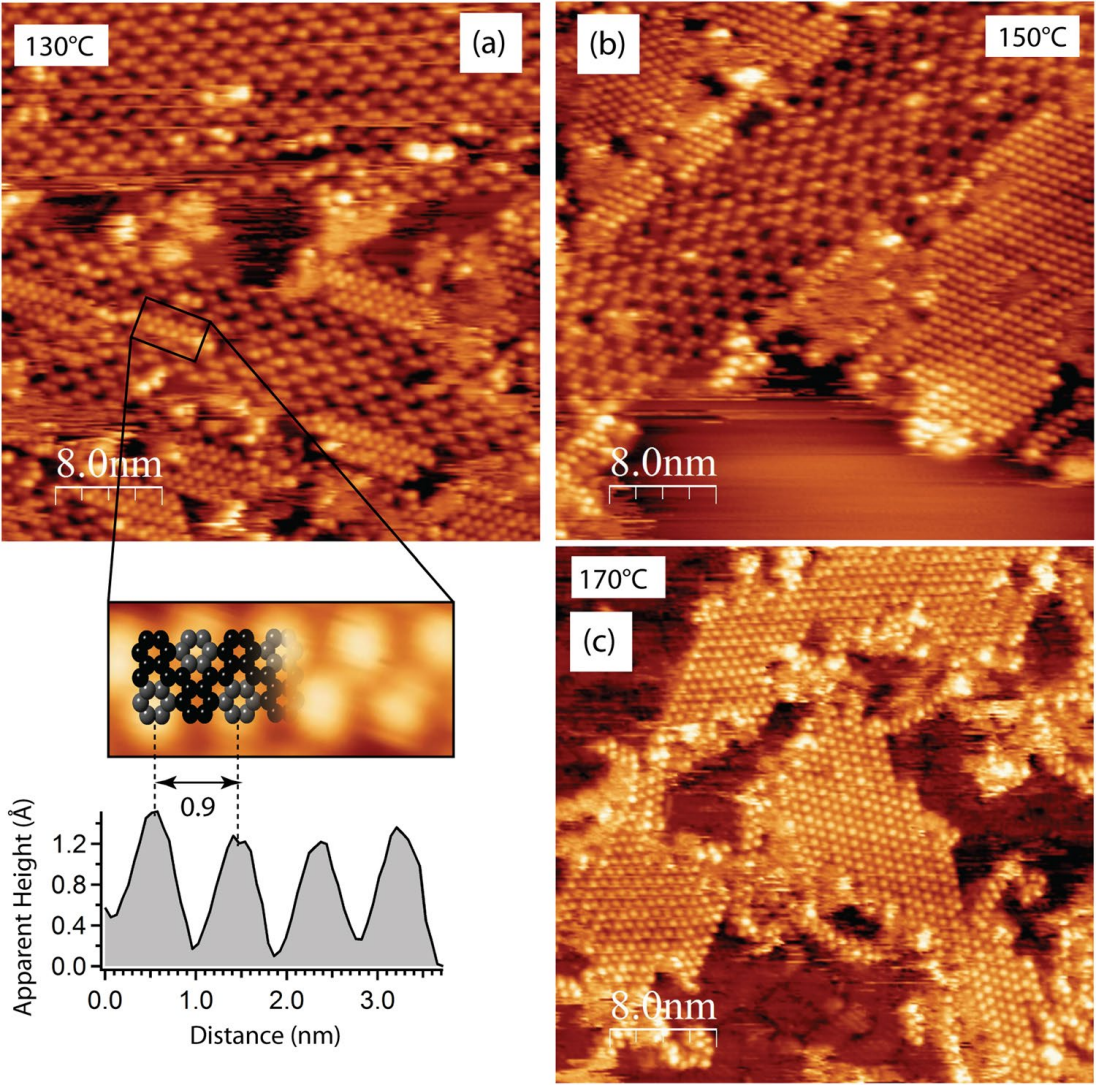
(**a**–**c**) STM images of OM chains on Ag(111) surface annealed for 30 min at the indicated temperatures. This set of STM images illustrates the transformation of OM chains into covalent ones. An enlarged region of the area indicated by the black rectangle is shown under (**a**). The height profile is taken along the chain axis and shows a periodicity of 0.9 nm characteristic for the polyanthracene chain, whose ball and stick model is overlaid on top. Grey colored carbon atoms show the lobes of the anthracene subunits pointing upward. Tunneling parameters (VS/IT): (**a**) +1.5 V/300 pA; (**b**) +1.7 V/100 pA; (**c**) +1.7 V/150 pA.

In order to verify the transformation from OM to covalent bonding and to determine the temperature threshold for this process, the sample with OM chains was subsequently annealed by additional 20 °C temperature intervals and characterized by STM at each step (Fig. 4a–c). Structural transformations are clearly visible in the STM images recorded at 130 °C (Fig. 4a). This is in good agreement with the C 1s XPS data (Fig. 2), indicating that dissociation of C–Ag bonds and formation of C–C bonds has started at this temperature. At 130 °C individual OM intermediates within the 2D island start to convert into dense chains with a visibly different structure. The chains are characterized by a “zigzag” pattern of periodic bright protrusions on either side of the central axis. As illustrated in the enlarged image of an individual chain and the corresponding line profile, they are characterised by a periodicity of 0.9 nm, which is close to the 0.85 nm expected from the structural model of the polyanthracene chain (*i.e*. individual DBBA molecules linked by covalent C–C bonds along the central axis) previously observed on Au(111)^15^. It should be noted that, as demonstrated by recent studies of GNR formation on the Cu(111) surface^32–37^, the polymer precursor for the formation of 7-AGNRs is difficult to distinguish from that preceding the growth of (3,1)-GNR by means of conventional STM. Due to the very similar periodicities of the chains and limited instrumental resolution in the STM, only imaging via a non-contact atomic force microscopy technique with a CO-functionalized tip was able to resolve the issue^36,37^. Nonetheless the proposed transformation from OM to polyanthracene chains agrees well with the fact that, according to refs^15,18^ and the results shown below, 7-AGNRs result from an on-surface Ullmann-type reaction for DBBA on Ag(111) surface. Moreover, unlike on the Cu(111) surface, where, to a large extent, the OM chains do not act as precursors for polymer chains, on Ag(111) a direct conversion of the OM chains into covalent chains can be followed with STM. Breaking of the OM bonds and the formation of covalent intermolecular connections begins within the short segments of the OM chains (Fig. 4a–c). The bonds at each end of the newly formed polymer chain appear to be either terminated by residual hydrogen present on the surface or bonded to the substrate. Logically, a polymerization produces a longitudinal contraction of the OM chain, which results in small vacant regions that are usually visible at the ends of polymer chains incorporated into a OM island. The resulting polyanthracene chains possess the same orientation with respect to the substrate as their OM precursor, which additionally demonstrates their structural affinity.

Annealing at higher temperatures promotes further C–C coupling reactions. Few polymer chains are visible at 130 °C; they appear in approximately equal numbers as the OM chains at 150 °C (Fig. 4b), while annealing at 170 °C completes the conversion of OM to covalent bonds (Fig. 4c). The resulting islands of polymer chains are surrounded by some (often considerable) amounts of individual disordered molecules, which are not incorporated in covalent chains. In summary, conversion of OM to polyanthracene chains can occur on Ag(111) while on the more active Cu(111) substrate this conversion is blocked due to a surface-assisted dehydrogenative coupling reaction^36,37^.

**Figure 5:**
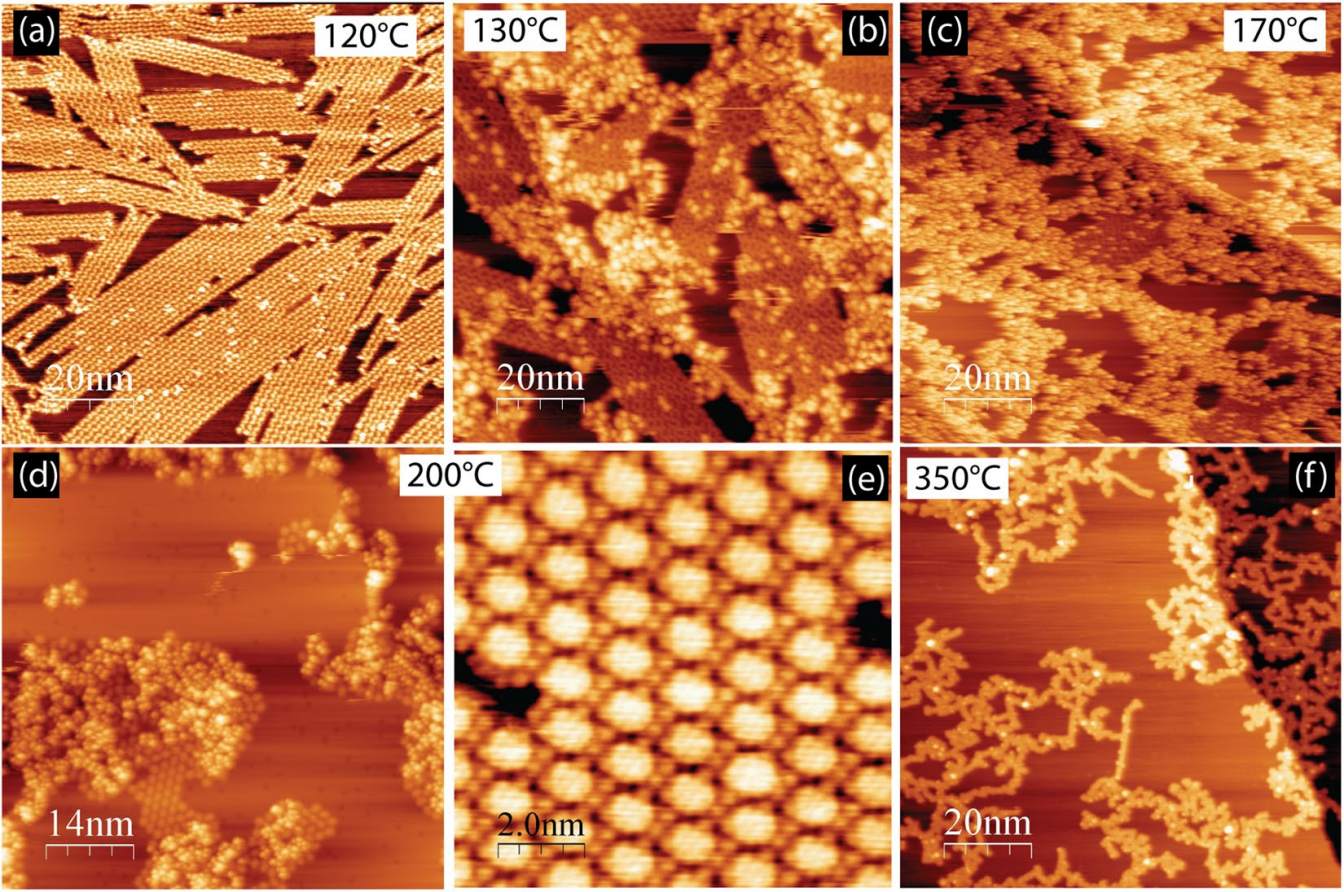
(**a**–**f**) STM images obtained after annealing DBBA on Ag(111) at indicated temperatures during the experiment when C-Ag-C bonds were not able to convert into the covalent ones. (**e**) The high-resolution image of the hexagonal island of nanographenes (square units) coordinated by Br adatoms (small round features in-between nanographenes. Tunneling parameters (V_S_/I_T_): (**a**) −1.7 V/200 pA; (**b**) +1.5 V/200 pA; (**c**) +1.5 V/200 pA; (**d**) +1.5 V/100 pA; (**e**) −0,04 V/230 pA; (**f**) −1.5 V/200 pA.

It should be noted that OM chains on Ag(111) are very fragile and it is not always possible to transform them into covalent ones. An example of the outcome of one such experiment is given in Fig. 5. In this case annealing induces cleavage of the C–Ag bonds, but molecules released from OM chains do not couple *via* C–C links (as can be seen already at 130 °C). The reasons behind this behaviour still remain to be explored in more detail, but it is suggested that immediately after cleavage of C–Ag bonds (which starts at 130 °C) the resulting unsaturated carbon bonds can be passivated by some active contaminant present on the surface. From Fig. 5(b,c) it can be seen that individual molecules released from the Ag atoms do not form polymer chains but prefer to assemble in disordered conglomerates with significantly larger apparent height instead. These disordered regions resemble those observed in the STM images of the 2,2′-dibromo-9,9′-bianthracene molecule on Ag(111)^35^ surface at RT and therefore it is proposed that they are passivated molecular radicals. The most probable reason is hydrogen, which is always present in vacuum chambers as the residual H_2_ gas and can possibly dissociate on the Ag(111) surface at elevated temperatures (especially in the presence of silver adatoms, nano-clusters, atomic steps, etc). In turn, atomic hydrogen is very chemically active and can saturate the newly formed dangling bonds thus blocking the formation of polymer chains. Annealing of such sample at 200 °C can induce flattening of some DBBA units which results in the formation of fused square fragments (nanographenes) assembled in hexagonal islands where the nanographenes are coordinated by Br atoms (Fig. 5d,e). Similar structures were observed for DBBA on Ag(111) in ref.^18^ and interpreted as an intermediate stage of the 7-AGNR formation. Nevertheless, as can be seen from Fig. 5(f), annealing at 350 °C leads to the formation of irregular dendritic structures. These structures appear as a product of the surface-assisted dehydrogenative coupling reaction between nanographenes, similar to those recently grown from the chlorine-substituted analogue of the DBBA molecule on Au(111).^57^ Interestingly, a small admixture of short GNRs can sometimes be seen in the STM images.

**Figure 6:**
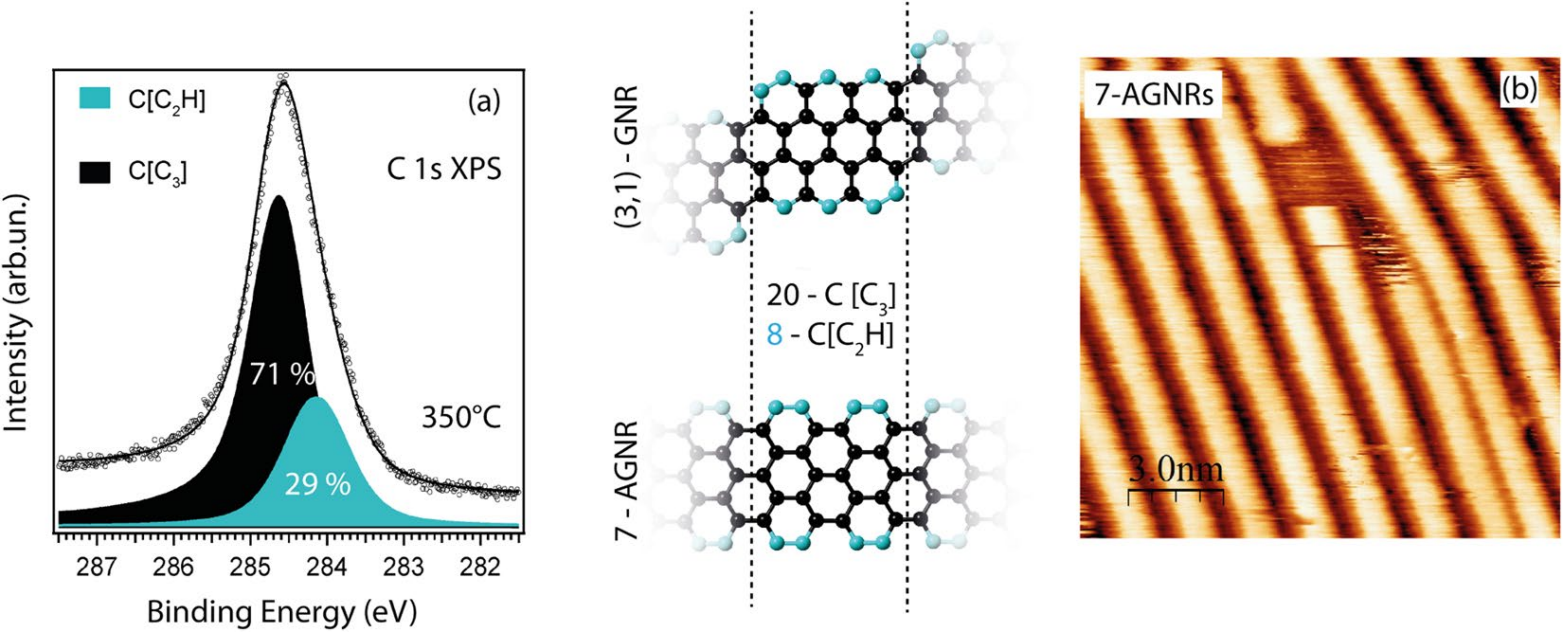
(**a**) C 1s XPS spectrum obtained after 350 °C annealing of polyanthracene chains on Ag(111) for 30 min. The photoemission peak is fitted in accordance with the ratio between the amounts of carbon atoms in chemically non-equivalent positions expected for both of the structural models shown to the right. The photon energy is 380 eV. (**b**) STM image which illustrates the resulting 7-AGNRs. Tunneling parameters (VS/IT): +1 V/500 pA.

Unlike nanographenes, further annealing of polyanthracene chains activates cyclodehydrogenation within the individual chains and the formation of graphene nanoribbons, similar to the process observed at the Au(111) surface and initially suggested by Cai *et al*.^15^. For Ag(111) annealing at 350 °C was sufficient to complete the cyclodehydrogenation process. The corresponding C 1s XPS and STM data are shown in Fig. 6(a) and (b), respectively. Similar to the C 1s XPS spectrum of 7-AGNRs on Au(111)^30,31^, the C 1s XPS peak of GNRs on Ag(111) can be fitted with two components, representing carbon atoms in C[C_3_] and C[C_2_H] configurations. The contributions of the individual components to the total C 1s signal, determined from the peak fitting analysis, are in agreement with the values expected from the model of a hydrogen-terminated 7-AGNR. It should be noted that an analysis of the XPS line profile of the C 1s core level alone cannot conclusively prove the formation of 7-AGNRs. As can be seen from the inset to the right of Fig. 6(a), the same spectral line shape is also expected for chiral (3,1)-GNR, which grows on the Cu(111): for both types of GNRs the repetition units have 20 carbon atoms in C[C_3_] sites and 8 carbon atoms in C[C_2_H] positions. Nevertheless, in agreement with refs^15,18^, the STM image in Fig. 6(b) unambiguously illustrates the formation of 7-AGNRs on the Ag(111) surface. As additionally shown in Fig. S4, 7-AGNRs can merge with each other longitudinally, and the STM images demonstrate electron scattering patterns characteristic of armchair graphene nanoribbons^18^.

## Discussion

The results described above elucidate the growth mechanism of 7-AGNRs on Ag(111), indicating that similar to DBBA on Cu(111), debromination of DBBA units is followed by the formation of OM chains. The structure of chains was identified from STM images and is supported by the DFT calculations. It is shown that unlike OM chains on Cu(111), the OM chains on Ag(111) can be converted into polyanthracene oligomers via an Ullmann-type reaction. Alternatively, if the formation of polymer chains is blocked, nanographene units can be formed as a result of cyclodehydrogenation within individual molecular units. Further annealing of polymer chains yields 7-AGNRs, while surface-assisted dehydrogenative coupling reaction between nanographenes leads to the formation of the disordered structures. Taking this into account, we can analyse the possible impact of the OM intermediates on the formation of GNRs on Ag(111) and Cu(111) from DBBA precursor molecules.

After the complete debromination of DBBA on both Ag(111) and Cu(111), the radical carbon atoms are saturated with metal adatoms as the OM chains are formed. This processes take place around 120 °C on Ag(111) and at RT on Cu(111). On the Ag(111) the Ullmann reaction is feasible and further annealing of OM chains in the 130 °C to 170 °C range leads to dissociation of OM bonds and formation of the covalent C–C bonds. As a result, polyanthracene chains can be formed. In contrast, on the Cu(111) the surface-assisted dehydrogenative coupling reaction inhibits the Ullmann coupling reaction^36,37^.

There are several reasons for such an effect. Firstly, our calculations indicate that the binding energies of a single metal atom to the debrominated DBBA unit are 2.40 eV for Ag and 3.07 eV for Cu. Therefore, in agreement with previous reports^45,50^, the OM bonds on silver are weaker than on copper. Secondly, to further discriminate between the reactivities of the two metal surfaces, kinetic processes such as on-surface diffusion have to be taken into account. It is logical to assume that weaker molecular-metal interaction increase the molecular diffusion ability on the silver surface. The critical role of diffusion for the formation of GNRs was recently demonstrated by C. Bronner *et al*. in their study of iodine- and bromine- containing molecules on the Au(111) surface^38^. It was shown that even though dehalogenation of iodinated monomers occurs on gold at lower temperature, the surface-stabilized monomers do not start polymerisation right away due to their reduced diffusivity. Taking into account the weaker molecule-metal interaction on the silver surface, the temperature for activation of the diffusion of molecular radicals is expected to be lower than on the copper surface. Thirdly, the temperature required to activate the surface-assisted dehydrogenation of hydrocarbons is significantly larger on Ag(111) than on Cu(111)^50,55,56^. Altogether this explains the ability of OM chains on Ag(111) to be converted into the polyanthracene ones without exceeding the surface stability of the molecular monomers. At the same time on Cu(111) dissociation of the carbon-metal bonds within the organometallic chain occurs in parallel with the surface-assisted dehydrogenative coupling^36,37^.

## Conclusions

We have demonstrated that the growth of GNRs on Ag (111) from a DBBA precursor molecule proceeds through the formation of OM chains close to 120 °C. Similar to the OM chains on Cu(111), these chains are composed of debrominated DBBA units connected *via* linking Ag atoms. The structure of OM chains on Ag(111) was modelled with DFT and is in agreement with the STM data. It was experimentally confirmed that the growth of 7-AGNRs on Ag(111) from DBBA precursor molecules proceeds via an Ullmann-type reaction. In general, the weaker C–Ag bonds (in comparison with the C–Cu bonds) reduce the diffusion barrier for molecular intermediates on silver, and the higher temperature required for the surface-assisted dehydrogenation on this surface makes the formation of polyanthracene precursors for 7-AGNRs *via* an Ullmann-type reaction feasible and proceeds on annealing at 170 °C. In contrast, on Cu(111) at temperatures close to 100 °C, dehydrogenative covalent coupling due to the surface-catalysed, selective dehydrogenation occurs in parallel with cleavage of the stronger C–Cu bonds. Therefore, while OM intermediates obstruct an Ullmann reaction between DBBA molecules on the Cu(111) substrate, they are directly involved in the formation of polyanthracene chains and, therefore, armchair GNRs from DBBA on Ag(111). As an alternative, the formation of flat nanographene units from OM chains is possible, but no 7-AGNRs can be subsequently grown in this case.

In general, our study contributes to the understanding of the role of OM intermediates in the bottom-up synthesis of nanostructures and underlines the importance of the multitechnique approach for investigation of the reaction mechanism.

## Methods

### Experimental methods

The temperature evolution of the DBBA/Ag(111) system upon controlled annealing was monitored by XPS and STM. Spectroscopic data were obtained at the D1011 beamline, MAX IV (Lund, Sweden). STM images were acquired using the MAX IV laboratory STM system (VT STM XA, Omicron Nanotechnology GmbH). The Ag(111) single crystals were cleaned by several cycles of Ar^+^ sputtering (E_Ar_^+^ = 1 keV) at RT and subsequent annealing in UHV at 550 °C. The cleanliness of the substrate was verified either by XPS and low-energy electron diffraction (LEED) or directly by STM. After an initial purification the DBBA molecules (Angene Chemical 99% purity) were deposited by sublimation under UHV conditions from a home-build Knudsen-cell onto the clean substrate maintained at either liquid nitrogen (LN) temperature (−140 °C on the sample), at RT or at 100 °C. The amount of deposited material was checked by XPS and STM and was less than a monolayer. The surface was gradually heated in UHV to monitor the evolution process.

Photoelectron spectra were measured in the normal emission geometry and normalized to the current in the storage ring and number of scans. The resolution of the SES-200 electron analyzer was 125 meV for the C 1s and Br 3d photoelectron spectra. The peak-fit analysis of the C 1s XPS spectra was performed with the *FitXPS* software^63^. The base pressure during spectroscopic measurements was better than 5 × 10^−10^ mbar. STM measurements were performed at LN temperature in an analysis chamber with a base pressure of 5 × 10^−11^ mbar. STM images were recorded in constant current mode using an electrochemically etched polycrystalline tungsten tip. The voltage, V_S_, corresponds to the sample bias with respect to the tip. The *WSxM* software was used for the processing of STM images^64^.

### Computational details

The calculations were carried out using DFT with the gradient-corrected exchange-correlation functional of Wu and Cohen (WC)^65^ as implemented in the SIESTA package^66^. The WC functional provides an adequate description of the lattice constants, crystal structures, and surface energies of solids and systems with layered structures. For example graphene or hexagonal boron-nitride (h-BN) monolayers deposited on 3d, 4d, and 5d transition-metal surfaces^58,67,68^. Double-ζ plus polarization function (DZP) basis sets were used to treat the valence electrons of all atoms, while the core electrons were represented by Troullier-Martins norm-conserving pseudopotentials^69^. Periodic boundary conditions were used for all systems, including the free molecules.

The Cu and Ag fcc lattices were optimized using the Monkhorst-Pack^70^ 14 × 14 × 14 k-point mesh for Brillouin zone sampling. The calculated lattice parameters for Cu (a = 3.625 Å) and Ag (a = 4.129 Å) are in a good agreement with the experimental values of a = 3.61496 Å and a = 4.0862 Å, respectively^71^. The optimized lattice of bulk Cu was used to construct a four-layer 9 × 9 slab for Cu(111) and 8 × 8 slab for Ag(111) surfaces, containing 324 and 256 metal atoms, respectively. The periodically replicated slabs were separated by a vacuum region of 20 Å. In the calculations the bottom two metal layers were fixed, while all other atoms are fully relaxed. Only the Γ point was used for sampling the Brillouin zone of the slabs because of the large size of the supercell. An energy cutoff of 200 Ry was chosen to guarantee convergence of the total energies and forces. A common energy shift of 10 meV was applied. Self-consistency of the density matrix was achieved with a tolerance of 10^−4^. For geometry optimization, the conjugate-gradient approach was used with a threshold of 0.02 eV Å^−1^.

To obtain the most stable configurations of the OM chains on Cu(111) and Ag(111) surfaces, a large number of starting configurations consisting of a stand-alone debrominated DBBA terminated with the corresponding metal atoms (up to 30 configurations of such molecular units for each surface) in different non-equivalent positions and orientations were generated. A similar approach was successfully used in our previous works on adsorption, structure optimization, and catalytic reactions of various types of molecules and metal clusters on surfaces^72–76^. As the next step, the most stable geometries of the stand-alone molecular units were used to form periodic chains on the metal surfaces, allowing different orientation, position, and periodicity order of molecular units with respect to each other. These configurations include two debrominated DBBA terminated with metal atoms per surface slab. The generated structures were further optimized in order to obtain the most stable OM chains.

### Data availability

The datasets generated and analysed during the current study are available from the corresponding author on reasonable request.

## Electronic supplementary material

Supplementary Information
