# Supplementary material for: Synthesis of armchair graphene nanoribbons from the 10,10′-dibromo-9,9′-bianthracene molecules on Ag(111): the role of organometallic intermediates
Source: Sci Rep. 2018 Feb 22;8:3506. doi: 10.1038/s41598-018-21704-3 (PMC5823938; doi:10.1038/s41598-018-21704-3)
Supplement: Supplementary file 1 — Supplementary Information [file 41598_2018_21704_MOESM1_ESM.pdf]

Supporting information for:

Synthesis of armchair graphene nanoribbons from the 10,10'-dibromo-9,9'-bianthracene molecules on Ag(111): the role of organometallic intermediates

K. A. Simonov,[1,2,3,*] A. V. Generalov,[2] A. S. Vinogradov,[3] G. I. Svirskiy,[3] A. A. Cafolla,[4] C. McGuinness,[5] T. Taketsugu,[6,7] A. Lyalin,[7] N. Mårtensson,[1] and A. B. Preobrajenski[2,*]

[1]Department of Physics and Astronomy, Uppsala University, Box 516, 75120 Uppsala, Sweden.
[2]MAX IV Laboratory, Lund University, Box 118, 22100 Lund, Sweden.
[3]V.A. Fock Institute of Physics, St. Petersburg State University, 198504 St. Petersburg, Russia.
[4]School of Physical Sciences, Dublin City University, Dublin 9, Ireland.
[5]School of Physics, Trinity College Dublin, College Green, Dublin 2, Ireland.
[6]Department of Chemistry, Faculty of Science, Hokkaido University, Sapporo 060-0810, Japan.
[7]Global Research Center for Environment and Energy Based on Nanomaterials Science (GREEN), National Institute for Materials Science (NIMS), Tsukuba 305-0044, Japan.
* *konstantin.simonov@physics.uu.se*
* *alexei.preobrajenski@maxlab.lu.se*

Additional STM characterization of DBBA on Ag(111) at room temperature

Two co-existing self-assembled structures formed by DBBA molecules on Ag(111) at RT are illustrated in Fig. S1(a-c). The arrangement of the DBBA units in dense molecular islands shown in Fig. S1(a) prevails above 30 ˚C, while the domains composed of the "flower"-like hexamer features in Fig. S1(c) tend to form at slightly lower temperatures, hence indicating a low energy barrier for the structural rearrangement of the system. Self-assembly of DBBA molecules on the Ag(111) surface was investigated in the recent paper by Shen et al.[1] Nevertheless, partial debromination of DBBA molecules at RT was not taken into account by the authors. For the half-debrominated DBBA molecules the "one-legged" adsorption geometry, similar to the one for the half-debrominated DBBA on Cu(110)[2] , can be suggested. This implies that molecular units tilted at various angles with respect to the surface can be imaged in the STM differently. Moreover, by analogy with previous studies of bromine-containing molecules on metal surfaces[2,3], Br atoms detached from the DBBA units can appear in the STM images as additional features surrounding the molecules.

In agreement with work of Shen et al.[1], dense molecular islands shown in Fig. S1(a) are most likely formed by the molecular rows (indicated by white lines), which lie side-by-side being regularly separated by the gaps due to the presence of dissociated bromine or due to relief of the strain in the molecular domain. Three types of domains in which DBBA rows align in three directions oriented at 120˚ to each other can be obtained for such molecular islands (Fig. S1c). The orientation of DBBA rows constituting

each domain is governed by the interaction with close-packed Ag(111) surface and hence the domains are rotated by 120 ˚ with respect to each other. The ordered islands co-exist with some admixture of disordered phase, which is clearly seen close to the domain boundaries.

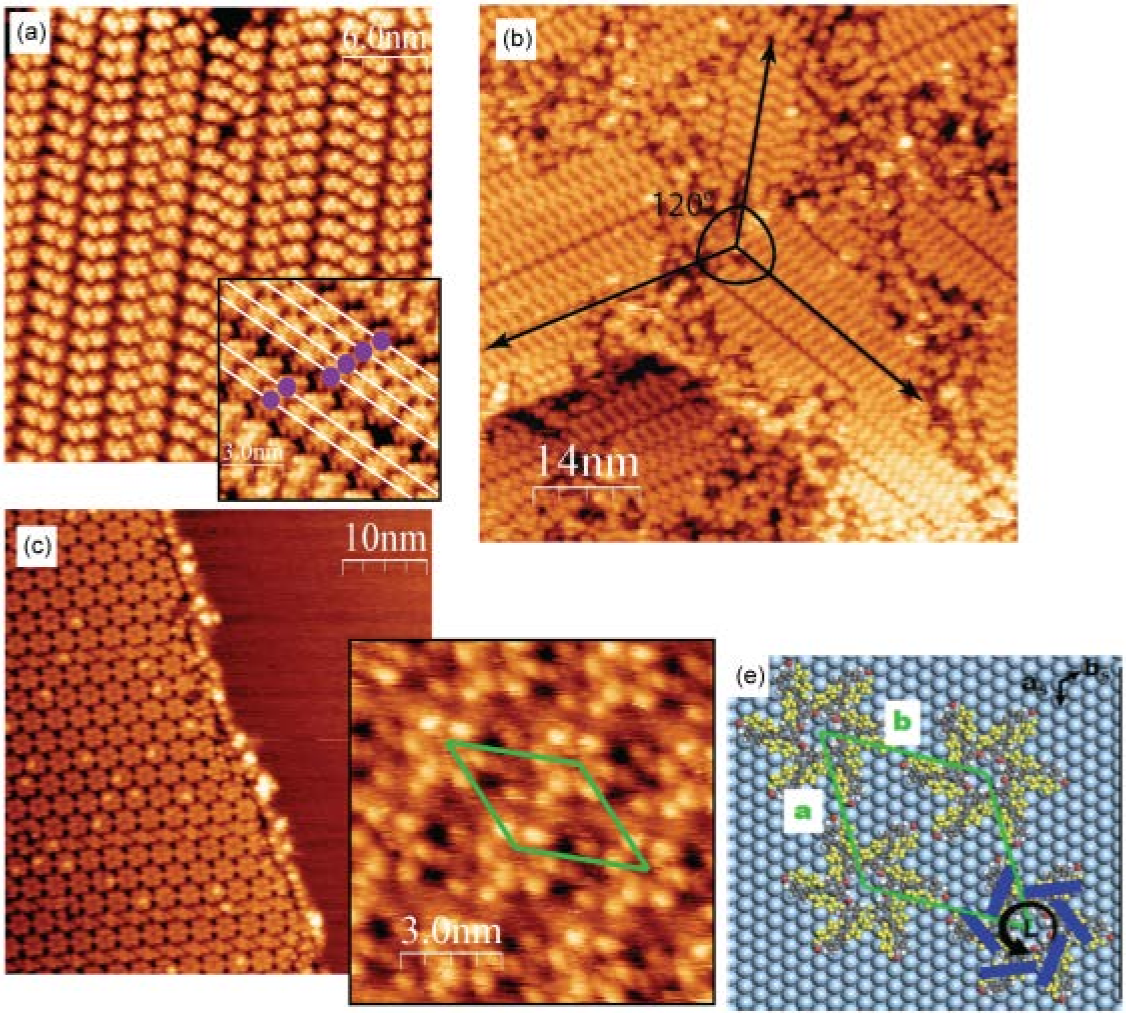

Figure S1 (a,c) STM images of different arrangements obtained for DBBA on Ag(111) at RT. Insets show the small-scale STM images of the corresponding areas. The violet circles in (a) indicate suggested positions of the DBBA molecules (see text for explanation). (b) STM image illustrating three types of domains (at 120˚ to each other) characteristic for the dense molecular islands obtained for DBBA on Ag(111) at RT and shown in (a). The disordered regions separating different domains are clearly visible. Tunneling parameters (VS/IT): (a) + 1.75 V/190 pA; (b) + 1 V/500 pA; (c) + 2 V/200 pA. (e) The structure of the hexamer phase shown in (c) as suggested in Ref. 1. *Adapted with permission from. Shen, Y. et al. Langmuir 33, 2993-2999 (2017). Copyright 2017 American Chemical Society.*

As for the hexamer phase (Fig. S1c), according to Ref.1, each hexamer is composed of six molecules (Fig. S1e). Since in Ref. 1 it was not taken into account that the molecules are half-debrominated, we can now assume that the molecules are pinned to the silver surface atoms on their side which is directed to the center of hexamer.

Organometallic chains and self-assembly of DBBA on Ag(111)

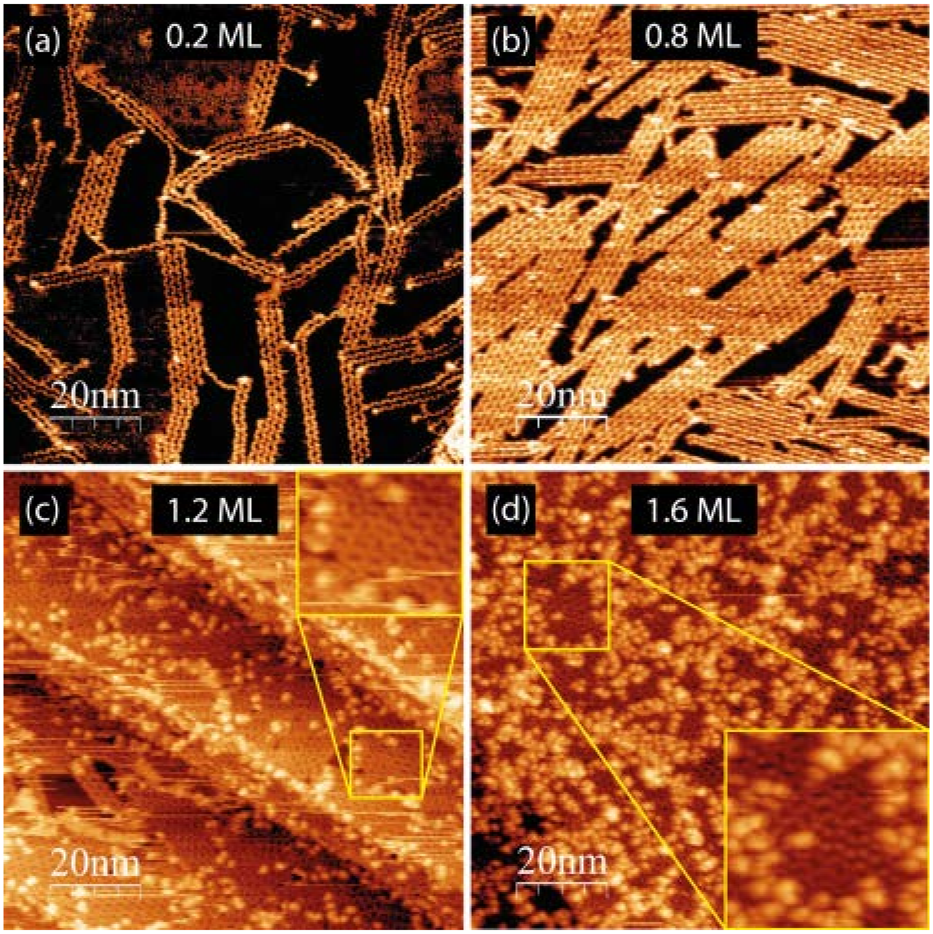

Figure S2. STM images showing OM chains at different coverage defined in monolayers (ML). The definition of the monolayer is area-based. Tunneling parameters (V_S/I_T): (a) - 1.7 V / 350 pA; (b) - 1.5 V / 300 pA; (c) + 1.5 V / 200 pA; (d) - 1.7 V / 200 pA.

Fig. S2 illustrates the islands of OM chains obtained after deposition of DBBA molecules on the Ag(111) surface kept at 100 ˚C. It can be seen, that OM chains can be formed at different coverages which excludes the effect of coverage on the appearance of OM chains suggested previously[1]. Moreover, Fig.3 shows the typical STM images obtained from the sample prepared by deposition of DBBA on Ag(111) at RT (Fig. S3a) followed by the annealing at 100˚C (Fig. S3b). It can be seen that islands of OM chains appeared instead of the hexamer phase as a result of the complete debromination of DBBA molecules.

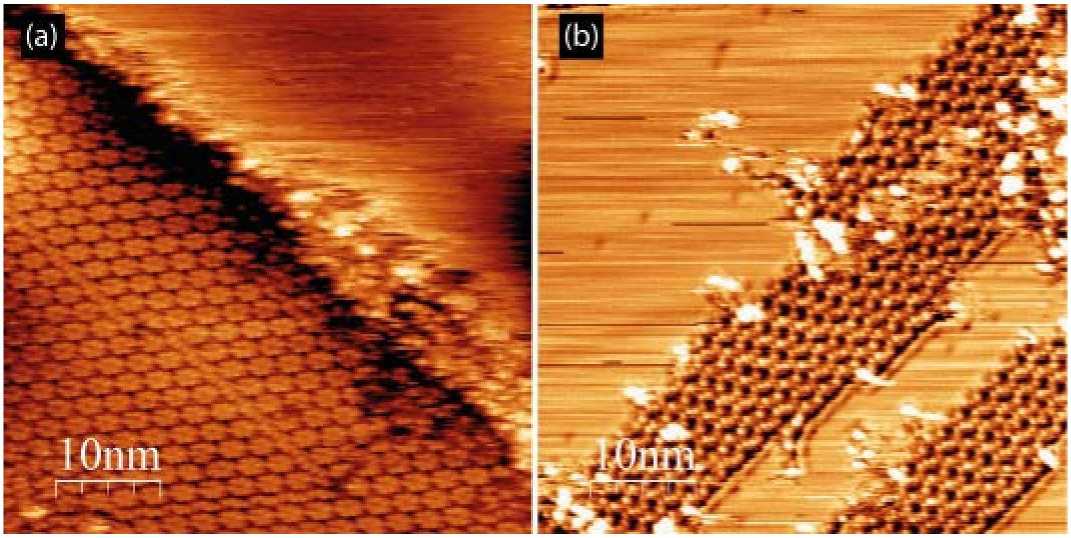

Figure S3. STM images recorded (a) after deposition of DBBA molecules at RT, and (b) after annealing of the same sample at 100˚C. Tunneling parameters (V_S/I_T): (a) + 1.5 V / 100 pA; (b) + 1.5 V / 200 pA.

STM characterization of AGNRs on Ag(111) surface

The STM image of GNRs/Ag(111) obtained at RT after annealing of the polyanthracene chains at 350 ˚C is shown in Fig. S3. In agreement with the STM studies of 7-, 14- and 21-AGNRs performed in Ref.3, the 7-AGNRs and 14-AGNRs (two 7-AGNRs merged together) shown in Fig. S3 demonstrate the characteristic electron scattering patterns unique for the particular type of GNR. Electron scattering patterns arise from the intervalley electron backscattering at armchair edges, and appearance of the patterns identical to those shown in Fig. 3 of Ref. 3 unambiguously proves the formation of graphene nanoribbons with armchair edge structure in our experiment.

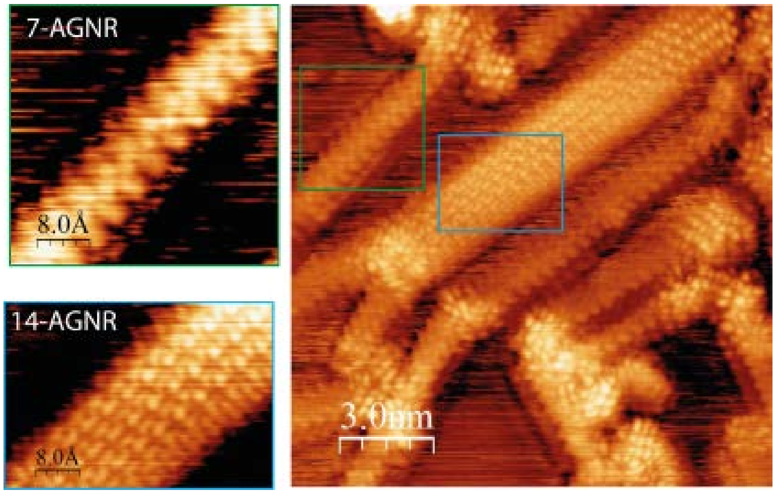

Figure S4. STM image showing electron scattering patterns in 7-AGNR and 14-AGNR grown on Ag(111) surface. Tunneling parameters (V_S/I_T): + 0.05 V / 950 pA.

Density of States (DOS) calculations for OM chains on Ag(111) and Cu(111)

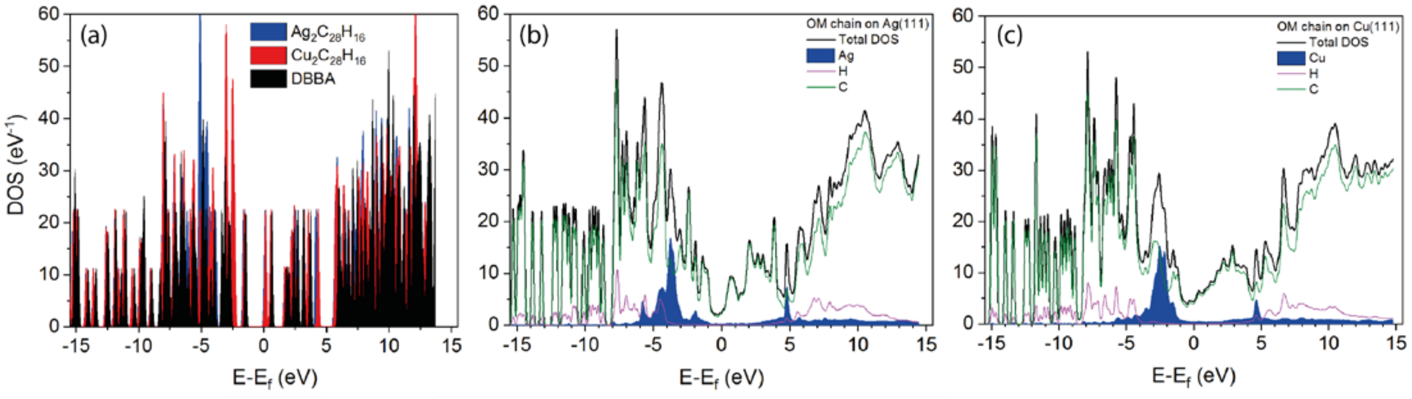

Figure S5. (a) Density of states (DOS) calculated for DBBA and metal-substituted molecular units; (b),(c) atom-projected DOS calculated for OM chains on Ag(111) and Cu(111). The corresponding calculated structures are presented in Fig 3e,f of the main manuscript.

The results of DOS calculations for various OM intermediates are presented in Fig. S5. From Fig. S5(a) one can clearly see that HOMO – LUMO gap decreases when Br is substituted with the M (M = Ag, Cu) in the stand-alone molecular unit isolated from the substrate. Moreover, additional metal-dominated states appear below and above the $E_{f.}$ Fig. S5(b) and Fig. S5(c) illustrate the DOS projected on M,C and H atoms in the OM chain on Ag(111) and Cu(111), respectively. The calculated DOS correspond to the two kinds of OM chains presented in Fig. 3 (e,f). For both OM chains the band gap is vanished as a result of their interaction with the metallic substrate. Moreover, interaction of molecular units with bridging metal atoms is responsible for the appearance of the additional sharp peak in the valence region (Fig. S5b,c). This characteristic feature can be used as an indication of the formation of organometallic bonds.

OM chains on Ag(111) and Cu(111)

As mentioned in the manuscript, when optimizing the structure of OM chains on Ag(111) and Cu(111) surfaces, the periodic boundary conditions were used. In this case, it is not individual isolated OM chain which is considered, but the periodic array of OM chains (see Fig. S6)

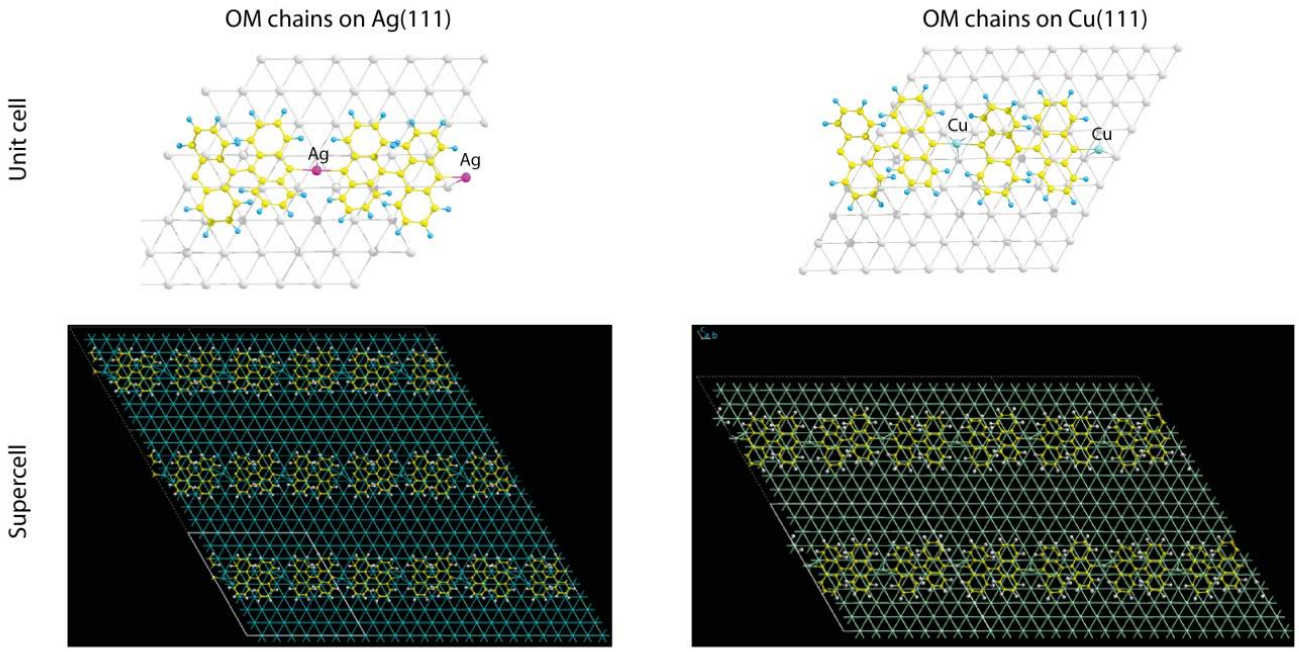

Figure S6. First row – Unit cells used in DFT calculations of OM chains on Ag(111) and Cu(111) optimized using the periodic boundary conditions. Second row – 3×3 and 2×3 supercells illustrating the array of unidirectional OM chains on Ag(111) and Cu(111) surfaces, respectively.

References

1 Shen, Y., et al. Chiral self-assembly of nonplanar 10,10′-dibromo-9,9′-bianthryl molecules on Ag(111), *Langmuir* **33**, 2993 (2017).

2 Simonov, K. A., et al. From graphene nanoribbons on Cu(111) to nanographene on Cu(110): critical role of substrate structure in the bottom-up fabrication strategy, *ACS Nano* **9**, 8997 (2015).

3 Simonov, K. A., et al. Comment on "Bottom-up graphene-nanoribbon fabrication reveals chiral edges and enantioselectivity", *ACS Nano* **9**, 3399 (2015).

4 Huang, H., et al. Spatially resolved electronic structures of atomically precise armchair graphene nanoribbons, *Sci. Rep.* **2**, 983 (2012).

5 Sánchez-Sánchez, C., et al. Purely armchair or partially chiral: noncontact atomic force microscopy characterization of dibromo-bianthryl-based graphene nanoribbons grown on Cu(111), *ACS Nano* **10**, 8006 (2016).

6 He, Y., et al. Fusing tetrapyrroles to graphene edges by surface-assisted covalent coupling, *Nat. Chem.* **9**, 33 (2017).
